# Mitochondrial metagenomics reveal the independent colonization of the world’s coasts by intertidal oribatid mites (Acari, Oribatida, Ameronothroidea)

**DOI:** 10.1038/s41598-024-59423-7

**Published:** 2024-05-21

**Authors:** Tobias Pfingstl, Shimpei F. Hiruta, Satoshi Shimano

**Affiliations:** 1 Department of Biology, University of Graz, Universitätsplatz 2, 8010 Graz, Austria; 2 Center for Molecular Biodiversity Research, National Museum of Nature and Science, Amakubo 4-1-1, Tsukuba, Ibaraki 305-0005 Japan; 3 Science Research Center, Hosei University, Fujimi 2-17-1 Chiyoda-ku, Tokyo, 102-8160 Japan

**Keywords:** Evolution, Phylogeny, Perm-triassic, Extinction, Mitogenome, Evolution, Genetics, Zoology

## Abstract

Oribatid mites are an ancient group that already roamed terrestrial ecosystems in the early and middle Devonian. The superfamily of Ameronothroidea, a supposedly monophyletic lineage, represents the only group of oribatid mites that has successfully invaded the marine coastal environment. By using mitogenome data and nucleic ribosomal RNA genes (18S, 5.8S, 28S), we show that Ameronothroidea are a paraphyletic assemblage and that the land-to-sea transition happened three times independently. Common ancestors of the tropical Fortuyniidae and Selenoribatidae were the first to colonize the coasts and molecular calibration of our phylogeny dates this event to a period in the Triassic and Jurassic era (225–146 mya), whereas present-day distribution indicates that this event might have happened early in this period during the Triassic, when the supercontinent Pangaea still existed. The cold temperate northern hemispheric Ameronothridae colonized the marine littoral later in the late Jurassic-Early Cretaceous and had an ancient distribution on Laurasian coasts. The third and final land-to-sea transition happened in the same geological period, but approx. 30 my later when ancestors of Podacaridae invaded coastal marine environments of the Gondwanan landmasses.

## Introduction

Oribatid mites are tiny arachnids that usually dwell in terrestrial environments, especially in soil and litter, where they play an important role in decomposition, nutrient cycling, soil formation and aggregation^1^. Oribatida are an ancient group, they already roamed early terrestrial ecosystems in the Early and Middle Devonian (ca. 410–380 mya) based on fossil records^2–4^. Some researchers^5^ used divergence time estimation and even suggested that oribatid mites originated in the Precambrian (571 ± 37 mya). Presently, there are more than 11,000 known species world-wide and they can be found from the polar regions to the tropics^6^. Despite their extremely high diversity, very few species have been able to adapt to saltwater environments and lead a life as typical coastal organisms. Some of them, as for example *Haloribatula tenareae* or *Sphaerochthonius litoralis*^7,8^, are single species within larger typically terrestrial evolutionary clades, suggesting that these represent rather exceptional incursions into coastal habitats that have happened in relatively recent times^9^. The superfamily of Ameronothroidea, a group of approx. 130 species, on the other hand, are known as an almost exclusively marine associated taxon and thus are supposed to be the only larger evolutionary lineage of oribatid mites having successfully invaded the marine littoral^9,10^. This unique group comprises five families, the Ameronothridae, Fortuyniidae, Podacaridae, Selenoribatidae and Tegeocranellidae^9^. The Ameronothridae and Podacaridae are limited to coasts of polar and cold temperate zones, whereas the former only occur in the northern and the latter only in the southern hemisphere^9^. The Fortuyniidae and Selenoribatidae are restricted to shores of the tropics and subtropics, and the Tegeocranellidae also prefer warmer climates but they are limnic and have no association with the marine environment at all^9^.

The evolutionary history of Ameronothroidea has been a matter of numerous controversial debates and there is still no consent about their phylogeny. One theory suggests that Ameronothridae and Podacaridae represent a single taxon originating from a widespread terrestrial ancestor that inhabited cold and wet soils and a warming of the world’s atmosphere pushed these mites to cooler locations in each hemisphere, subsequent glaciation events finally forced them to occupy coastal areas^11^. Although the authors of this theory assumed a monophyletic origin of all Ameronothroidea, they did not explain or mention how the other warm-adapted families of this group could fit into this scenario. Another theory^12,13^ suggests that the invasion of coastal environments took place independently in three distinct latitudinal bands, with the Ameronothridae in the northern cold-temperate, the Fortuyniidae and Selenoribatidae in the tropical, and the Podacaridae in the southern cold-temperate region. Drivers for these marine association were supposedly glaciations in polar regions, i.e. ice sheets moving closer to the coast reduced inhabitable area and thus pushed the fauna closer to the coast, and competition in the subtropics and tropics, i.e. favorable conditions like warm temperatures and high humidity allowed many species to prosper but they had to compete for limited resources leading to the occupation of vacant niches^13^. Recent preliminary molecular genetic studies^14–16^ support the latter theory, and indicate that morphological similarities of certain ameronothroid marine associated groups are probably a result of convergent evolution and not of common origin^17^. Despite the growing evidence for an independent origin, Ameronothroidea are still given as monophyletic in the only existing and frequently used catalogue of oribatid mites of the world^6,18^. Indeed, molecular genetic studies are few and do not include enough taxa and markers to justify a large-scale change of long-standing systematics. High throughput DNA methods, like shotgun mitochondrial metagenomics (MMG), are promising approaches for phylogenetic analyses of species that are difficult to investigate with conventional morphological and molecular methods^19^. The mitochondrial genome is characterized by its circular topology, compact size (approx. 1.4 kbp), and the absence of introns, resulting in a high gene density^20^. Because of these characteristics, phylogenetic analysis uses not only the sequence, but also the arrangement of genes and their evolutionary patterns. Complete mitochondrial genomes are increasingly used as molecular markers for estimating phylogenies and, although not numerous, there have been studies on several mite taxa, as for example, house dust mites^21^, scabies mites^22^, oribatid mites^23,24^ and mites in general^19^.

In the present study, we analysed complete mitochondrial genomes of selected marine associated ameronothroid mites and possible terrestrial relatives to test which of the above given hypotheses could hold true. Additionally, we applied molecular dating techniques to estimate the geological periods in which land-to-sea transitions of oribatid mites could have happened. Knowing the approximate date for the invasion of coastal environments could give us important clues about paleoclimate and other factors that may have driven oribatid mites to colonize the world’s coastlines, and could allow us to finally settle the controversial debate about the evolutionary history of ameronothroid mites.

## Results

### Phylogeny and molecular dating

**Figure 1:**
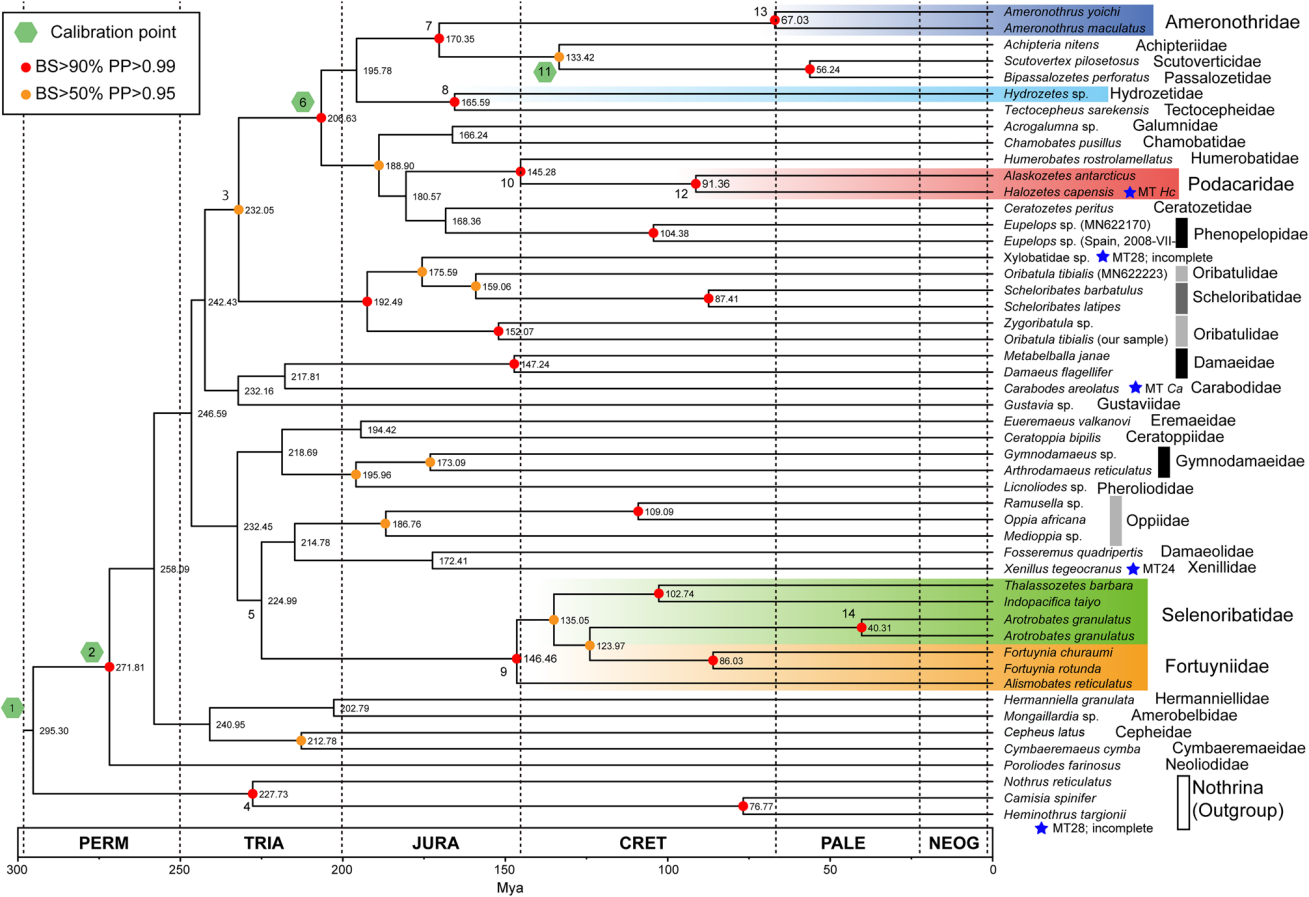
Time-calibrated tree inferred from mitochondrial 13 PCGs dataset. The colored circle on the node indicates high or moderate nodal support; the red circle BS > 90% and PP > 0.99; the orange circle BS > 50% and PP > 0.95. Each node has an estimated divergence time (mya). The nodes with the serial number (1–14) are detailed in Table 1; the nodes used for absolute time calibration are indicated as numbers enclosed with green hexagons. The five families of aquatic-adapted mites are highlighted. The OTUs with a different mitogenome gene arrangement from a general pattern for Brachypylina have a star symbol and Mitotype name. There are two *Arotrobates granulatus* species in the dataset, representing different populations from neighbouring southern Japanese islands (Table 2) and possibly a cryptic species complex^48^.

Using 13 PCGs (protein coding genes) in the mitochondrial genome, we constructed a maximum likelihood phylogenetic tree and estimated the divergence age using Bayesian methods (Fig. 1). A phylogenetic tree with 95% confidence intervals for the estimated divergence time on each node is also provided (Supplementary Fig. S1).

**Figure 2:**
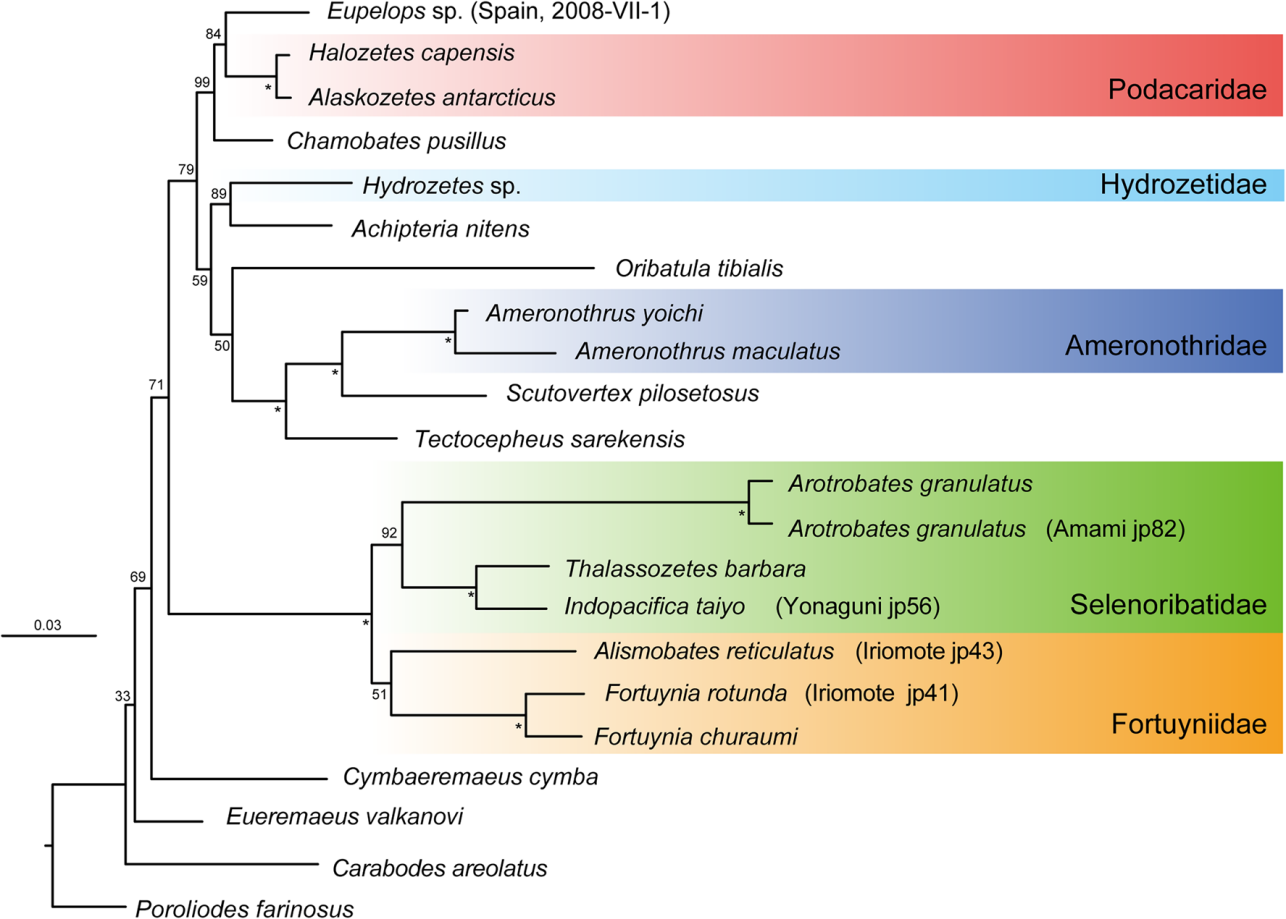
The Maximum likelihood tree inferred from the combined 18S, 5.8S, and 28S rRNA genes dataset. For each node with a BS value, asterisks indicate BS = 100%. The five families of aquatic-adapted mites are highlighted in colours.

As in previous studies, the monophyly of Brachypylina mites was strongly supported by our data, with the main Brachypylina lineages appearing by the end of the Triassic. In the ML tree based on the three nuclear rRNA genes (Fig. 2), the higher systematic relationship of Brachypylina was weakly supported, the same as 13 PCGs from the mitogenome (Fig. 1). However, these branches generally supported the lower systematic relationships of Brachypylina, and their resolution was high enough. The phylogenetic tree using three nuclear rRNA genes (Fig. 2) also showed four independent invasions of aquatic habitats. The Podacaridae were given as monophyletic group with the terrestrial *Eupelops* sp. as sister taxon and the freshwater aquatic *Hydrozetes* sp. clustered with the terrestrial *Achipteria nitens* with high bootstrap support. The Ameronothridae were placed as sister group to the terrestrial *Scutovertex pilosetosus* and *Tectocepheus sarekensis* with 100% bootstrap value support*,* and the Fortuyniidae and Selenoribatidae were given with very high support as monophyletic group and were placed far from the other aquatic adapted groups.

In the tree based on whole mitogenome data, the five families of aquatic-adapted mites were also divided into four independent lineages in the resulting tree (Fig. 1). The divergence times of all the lineages are estimated to have diverged before the Cretaceous period.

The freshwater-appearing *Hydrozetes* sp., made a clade with *Tectocepheus sarekensis* of the Tectocepheidae. The estimated divergence age was 165.59 mya with a 95% confidence interval of 124.08–197.74 mya (Table 1).

**Table 1:** Divergence times, statistical support of phylogenetic analysis for major nodes, and the node used for calibration in the molecular dating analysis.

Node	Note	Calibration point	Divergence time (mya)		Node support
			Median	95% HPD interval	
1	Brachypylina + Nothrina tMRCA	x	295.30	258.64–335.05	N.A
2	Brachypylina tRMCA	x	271.81	237.12–309.48	BS = 100, PP = 1.0
3			232.05	208.54–256.54	BS = 74, PP = 0.98
4	Nothrina tMRCA		227.73	166.54–299.52	BS = 100, PP = 1.0
5	Selenoribatidae + Fortuyniidae divergence		224.99	199.26–251.06	BS < 50, PP = 0.79
6		x	206.63	187.83–225.85	BS = 99, PP = 1.0
7	Ameronothridae divergence		170.35	114.43–173.17	BS = 94, PP = 1.0
8	*Hydrozetes* sp. and *Tectocepheus sarekensis* tMRCA		165.59	124.08–197.74	BS = 93, PP = 1.0
9	Selenoribatidae + Fortuyniidae tMRCA		146.46	113.98–178.93	BS = 100, PP = 1.0
10	Podacaridae divergence		145.28	114.43–173.17	BS = 100, PP = 1.0
11	Scutoverticidae divergence	x	133.42	106.19–160.26	BS = 88, PP = 1.0
12	*Halozetes capensis* and *Alaskozetes antarcticus* tMRCA		91.36	60.47–121.26	BS = 100, PP = 1.0
13	*Ameronothrus yoichi* and *A. maculatus* tMRCA		67.03	39.68–98.21	BS = 100, PP = 1.0
14	tMRCA for two *Arotrobates granulatus* sample		40.31	23.56–62.27	BS = 100, PP = 1.0

In node support, BS is a bootstrap value for the Maximum likelihood tree, and PP is a posterior probability for the Bayesian inference.

The lineages, occurring in the tidal environment, were divided into three major groups, one of which became a robust monophyletic group containing the two families Fortuyniidae and Selenoribatidae. However, the species of the two families were nested within each other, and each family did not become a monophyletic clade. Although not well supported, the lineage consisting of these two families is estimated to have diverged from its common terrestrial ancestor at 224.99 mya (95% HPD interval 199.26–251.06 mya), around the middle of the Triassic.

The two families Ameronothridae and Podocaridae, which are distributed in the coastal environment of each of the north and south polar regions were polyphyletic. Their estimated divergence ages from their terrestrial ancestors were 170.35 mya (95% HPD interval 114.43–173.17 mya) and 145.28 mya, respectively (Fig. 1, Table 1). The podacarid *Halozetes capensis*, also had a different mitochondrial gene arrangement than the other marine associated oribatid mites (Supplementary Fig. S2).

## Discussion

Certain authors^9,13^ already argued that monophyletic Ameronothroidea are an improbable hypothesis and preliminary molecular genetic studies^14–17^ supported this view. However, these studies did not include enough taxa as well as enough molecular genetic markers of all relevant ameronothroid groups to provide unequivocal evidence for their paraphyletic origin^17^. Present results are comprehensive enough to confirm that the superfamily of Ameronothroidea is a paraphyletic assemblage consisting of three distinct not closely related phylogenetic lineages. The first lineage consists of the subtropical and tropical families Fortuyniidae and Selenoribatidae, the second lineage is represented by the monogeneric and temperate northern hemispheric Ameronothridae, and the third lineage comprises the single family of cold-temperate southern hemispheric Podacaridae. These results corroborate the hypothesis of the marine associated lifestyle having evolved independently in three different latitudinal bands^9,13^.

**Figure 3:**
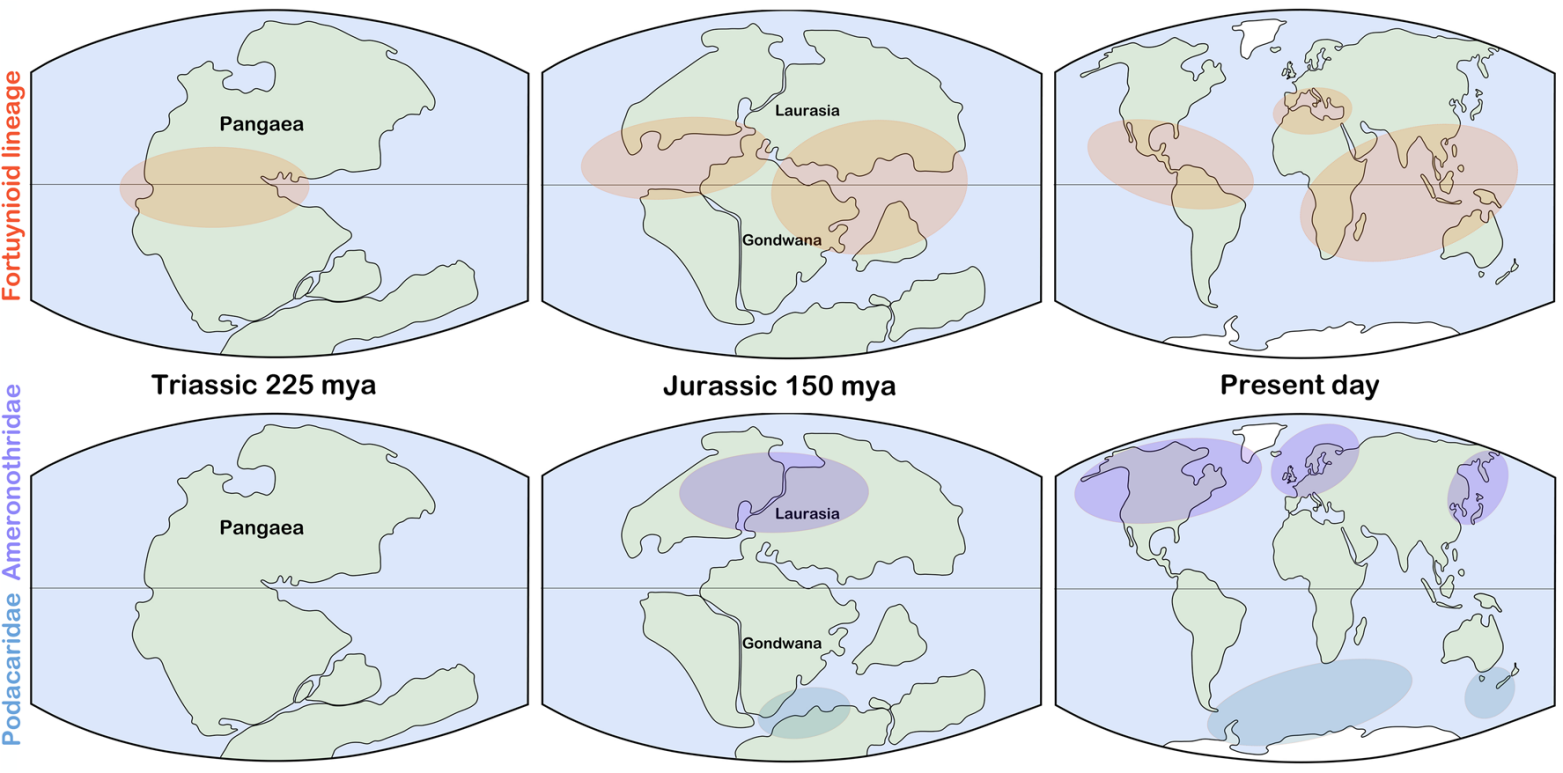
Historical biogeography of marine associated oribatid mite lineages. Coloured areas indicate distributional ranges of each group in the respective geological era. Dashed line represents equator. Fortuynioid lineage = Fortuyniidae + Selenoribatidae.

The ancestor of monophyletic Fortuyniidae and Selenoribatidae (from here referred to as ‘fortuynioid group’) was the first to colonize coastal environments. Molecular calibration of our phylogeny dates this oldest land-to-sea transition of oribatid mites sometime to the Triassic and Jurassic era (ca. 225–146 mya). The present-day distribution of fortuynioid taxa, with occurrences on coasts of each continent, except Antarctica^9^, indicates that common terrestrial ancestors of this lineage were widely distributed across Pangaea^25^ and thus suggests an early invasion of the coast in the Triassic when the supercontinent Pangea still existed (Fig. 3). However, a recent study^33^ included a few fortuynioid members and concluded that these diverged from the terrestrial *Charassobates* approx. 139 million years ago, which would date the land-to-sea transition of this group much later in the Cretaceous. Our results, on the other hand, suggest that the split between Fortuyniidae and Selenoribatidae happened sometime in the late Jurassic or early Cretaceous, which would mean that these mites already showed a very strong association with the marine environment during the Cretaceous. Mangroves are also suggested to have originated in the Cretaceous^34^ and many of the extant members of Fortuyniidae and Selenoribatidae are mangrove dwelling species. The adaptation to mangroves as habitats and their subsequent radiation could also have triggered a strong diversification of the mites in this period of time, as we see it in our phylogenetic tree. However, Pepato et al.^33^ dated the split between these two intertidal mite families to 75.5 million years ago which clearly contradicts our data. This age would be quite young considering the relatively high diversity of these groups, most family-level taxa within Acariformes were dated to the Jurassic and Triassic^19^ and thus would be older than the marine associated fortuynioid taxa. Fortuyniidae and Selenoribatidae are exclusive inhabitants of the intertidal environment and only feed on intertidal algae^9^, there is not a single species that can be found in terrestrial environments which argues for a relatively longer association with the marine environment. Moreover, in the Cretaceous Gondwana broke up and present-day continents began to form, in order to achieve the present-day distribution, with occurrences on all continents, except Antarctica, fortuynioid taxa would have had enormous dispersal potential, which is rather unlikely considering the small size and low mobility of these tiny organisms^1^. Based on these arguments, an earlier origin in the Triassic, as indicated by our data, seems more likely. The supposedly Pangean ancestors most likely developed aquatic adaptations to live in freshwater habitats and then colonized the littoral coasts. Such an evolutionary scenario is supported by the suggested close relationship of Fortuyniidae and Selenoribatidae to the freshwater limnic Tegeocranellidae^9,26^. A recent study^17^ further hypothesized these three families to form a monophyletic cluster with a common terrestrial origin. Transitions from land-to-sea are supposed to take place only when recipient environments are newly established, severely disturbed or populated by unspecialized species^27^. The Permian–Triassic boundary witnessed a significant and permanent ecological change resulting in a mass extinction event^28^. This end-Permian biodiversity crisis was marine centered^29^, experienced dysoxic to anoxic ocean environments^30^ and a global sea level rise^31^. The already impoverished marine biota became extinct and a wide range of coastal ecological niches became vacant. This vacant niche availability and change of competitive pressure may have facilitated the incursion of littoral environments by fortuynioid ancestors. The non-oribatid Halacaridae are purely marine mites that have secondarily invaded the sea early in the Permian era^32^ and the diversification of these mites intensified significantly after the Permian–Triassic extinction event^33^ which supports the assumption that many marine niches became available after this period.

According to our phylogeny, the Ameronothridae colonized the marine littoral later in the late Jurassic—Early Cretaceous period (ca. 170.35 mya), when the continents Laurasia and Gondwana existed (Fig. 3). This family is monogeneric and presently consists of 16 known species that are distributed in the northern hemisphere, on the North American continent, Europe and Eurasia^35^. This harmonic distribution pattern is indicative of a typical Laurasian fauna and suggests that common terrestrial ancestors of this family had an ancient distribution across former Laurasia^25^ which in turn supports the inferred timing of land-to-sea transition of this group. *Ameronothrus* species show various degrees of association with the littoral environment, i.e. some species are exclusively intertidal, others are transition species and one species, namely *A. lapponicus*, is purely terrestrial found only far inland^11^. This ecological variance, first, points to a more recent invasion of the coastal environment than their exclusively intertidal fortuynioid counterpart, and second, it indicates that the land-to-sea transition of Ameronothridae most likely happened directly, which means that terrestrial ancestors occurring near the coast probably began to browse on washed ashore algae and marine organic debris and subsequently adapted to the littoral environment^36^. Our phylogeny places the Ameronothridae close to the Licneremaeoidea and although morphology is controversial in this regard, ecology could give us an interesting clue. The licneremeoid *Scutovertex arenocolus* and *Scutovertex pilosetosus* are species that dwell exclusively in the supralittoral zone and thus are adapted to coastal environments^37^. Consequently, it is assumable that Ameronothridae and Licneremaeoidea share a common ancestor that showed a preadaptation to semiaquatic habitats allowing its descendants to invade the marine littoral.

Krause et al.^15^ argued that the aquatic freshwater lifestyle of Limnozetoidea evolved in convergence with the aquatic saltwater lifestyle of ‘Amerononothridae’ and we can confirm this assumption, as our tree based on mitogenome data places the limnozetid *Hydrozetes* sp. as sister taxon to the terrestrial Tectocepheidae with a divergence time of approx. 165 mya. The Ameronothridae also evolved their aquatic lifestyle in this geological era and it seems that Jurassic times generally favored the incursion of aquatic habitats by oribatid mites.

The Podacaridae represent the third evolutionary lineage that invaded the marine littoral environment independently. Our molecular calibration dates their origin to the late Jurassic–early Cretaceous (ca. 145.28 mya), which falls, more or less, into the same period when the Ameronothridae conquered Laurasian coastal environments (Fig. 3). Presently, there are four podacarid genera with ca. 24 species showing distributions from Antarctica, to all major sub-Antarctic Islands, to South Africa and New Zealand^38^. This specific southern hemispheric distribution points to a Gondwanan origin^25^, which is in contrast to the Laurasian Ameronothridae.

Podacaridae show an ecological variability similar to Ameronothridae, with species being exclusively intertidal, transitional or typical terrestrial^40^. There is yet no evidence of speciation from a terrestrial to a marine group or vice versa and it seems that a substantial ecological flexibility allowed the terrestrial and supralittoral species to invade the marine intertidal environment^39^. Glaciation has played a major role in shaping biotic systems in polar regions^41^ and has led to the extinction of many terrestrial taxa, with many other having moved to intertidal marine environments to escape the effects of ice scouring^13^. Podacarid Antarctic oribatid mites increase the glycerin concentration in their bodies to prevent freezing^42^. The inferred origin of these two families clearly predates these ice ages but the adaptation to cold-temperatures played an important role in the evolution of this group. Cold-temperature-adaptation traits may have facilitated the species to link the land-to-sea transition during the quaternary glaciations.

Our phylogeny places the Podacaridae in closest relationship to ceratozetoid species. There are hardly any morphological characters linking the two taxa and therefore such a relationship has not been considered seriously yet by any acarologist. However, immatures of most podacarid species show porose sclerites on their hysterosoma, which are being regarded as an apomorphy within the family^44^. Similar hysterosomal sclerites are present in juveniles of Ceratozetoidea^45^ but these were suggested to be analogous structures^44^. Finding the closest terrestrial relatives of Podacaridae needs further investigation and the inclusion of yet lacking members of all terrestrial superfamilies.

## Material and methods

### DNA sequencing

22 Brachypylina mites were collected and preserved in 99.5% ethanol (Table 2).

**Table 2:** Sample list with DDBJ/EMBL/GenBank accession numbers of nuclear rRNA genes for the phylogenetic reconstruction in supplement S1.

Family	Species	Nuclear rRNA	Habitat	Sampling date	Voucher	Locality	GPS point	References
Ameronothridae	*Ameronothrus yoichi*	LC817344	Tidal Arctic	14 Sep. 2018	NSMT-Ac: Xxxxx	Yoichi, Hokkaido, Japan	43° 14′ 52.1″ N 140° 42′ 35.1″ E	This study
	*Ameronothrus maculatus*	LC817345	Tidal Arctic	27 Sep. 2019	IBUG: Am-DE01	Bremen, Germany	53° 04′ 45″ N 08° 48′ 06″ E	This study
Achipteriidae	*Achipteria nitens*	LC817346	Soil	12 Aug. 2020	IBUG: An-CL07	Leechwald, Graz, Austria	47° 05′ 05″ N 15° 27′ 48″ E	This study
Scutoverticidae	*Scutovertex pilosetosus*	LC817347	Soil	27 Sep. 2019	IBUG: Sp-DE01	Bremen, Germany	53° 04′ 45″ N 08° 48′ 06″ E	This study
Hydrozetidae	*Hydrozetes* sp.	LC817348	Fresh water	2 Feb. 2017	IBUG: Hs-PA08	Rio Camatillo, Panama	09° 06′ 22″ N 79° 41′ 20″ E	This study
Tectocepheidae	*Tectocepheus sarekensis*	LC817349	Soil	12 Aug. 2020	IBUG: Ts-CL07	Leechwald, Graz, Austria	47° 05′ 05″ N 15° 27′ 48″ E	This study
Chamobatidae	*Chamobates pusillus*	LC817350	Soil	12 Aug. 2020	IBUG: Cp-CL01	Leechwald, Graz, Austria	47° 05′ 05″ N 15° 27′ 48″ E	This study
Podacaridae	*Alaskozetes antarcticus*	LC817351	Tidal Antarctic	7 Jun. 1979	NIPR: A02632	Fikdes Pen, King Gerorge Island, South Shetland Island, Antarctica	62° 12′ 55.7″ S 58° 57′ 46.8″ W	This study
	*Halozetes capensis*	LC817352	Tidal Antarctic	22 Feb. 2019	IBUG: Hc-ZA27	DeHoop, South Africa	34° 28′ 41″ S 20° 30′ 47″ E	This study
Phenopelopidae	*Eupelops* sp.	LC817353	Soil	1 Jul. 2008		Carrer de Mallorca, Barcelona, Spain	41° 24′ 10.0″ N 2° 10′ 24.0″ E	This study
Oribatulidae	*Oribatula tibialis*	LC817354	Soil	28 Aug. 2020	IBUG: Ot-CL12	Vorauer Schwaig, Styria, Austria	47° 30′ 41″ N 15° 57′ 24″ E	This study
Carabodidae	*Carabodes areolatus*	LC817355	Soil	12 Aug. 2020	IBUG: Ca-CL03	Leechwald, Graz, Austria	47° 05′ 05″ N 15° 27′ 48″ E	This study
Eremaeidae	*Eueremaeus valkanovi*	LC817356	Soil	12 Aug. 2020	IBUG: Ev-CL01	Leechwald, Graz, Austria	47° 05′ 05″ N 15° 27′ 48″ E	This study
Selenoribatidae	*Arotrobates granulatus*	LC817359	Tidal	21 Mar. 2019	IBUG: Ag-JP68	Okinawa-jima, Okinawa, Japan	26° 38′ 59″ N 127° 51′ 22″ E	This study
		LC817360	Tidal	24 Mar. 2019	IBUG: Ag-JP82	Amami-Oshima, Kagoshima, Japan	28° 08′ 50″ N 129° 18′ 20″ E	This study
	*Thalassozetes barbara*	LC817361	Tidal	28.Feb.17	IBUG: Tb-BA30	Bathsheba, Barbados	13° 12′ 53″ N 59° 31′ 27″ W	This study
	*Indopacifica taiyo*	LC817362	Tidal	18 Mar. 2019	IBUG: It-JP56	Yonaguni-jima Island, Okinawa, Japan	24° 26′ 19″ N 122° 58′ 20″ E	This study
Fortuyniidae	*Fortuynia churaumi*	LC817357	Tidal	22 Mar. 2019	IBUG: Fc-JP76	Ie no hama, Sosu, Okinawa-jima Island, Japan	26° 47′ 40.2″ N 128° 19′ 7.6″ E	This study
	*Fortuynia rotunda*	LC817358	Tidal	16 Mar. 2019	IBUG: Fr-JP41	Iriomote-jima Island, Okinawa, Japan	24° 19′ 17.43″ N 123° 54′ 38.87″ E	This study
	*Alismobates reticulatus*	LC817363	Tidal	16 Mar. 2019	IBUG: Ar-JP43	Iriomote-jima Island, Okinawa, Japan	24° 17′ 38.69″ N 123° 51′ 58.13″E	This study
Cymbaeremaeidae	*Cymbaeremaeus cymba*	LC817364	Soil	12 Aug. 2020	IBUG: Cc-CL05	Leechwald, Graz, Austria	47° 05′ 05″ N 15° 27′ 48″ E	This study
Neoliodidae	*Poroliodes farinosus*	LC817365	Soil	12 Aug. 2020	IBUG: Pf-CL04	Leechwald, Graz, Austria	47° 05′ 05″ N 15° 27′ 48″ E	This study
Passalozetidae	*Bipassalozetes perforatus*		Soil		BMNH:1427542	Sierra de Grazalema, El Bosque, Spain	36° 44′ 54.0″ N 5° 29′ 25.7″ W	^19^
Galumnidae	*Acrogalumna* sp.		Soil		BMNH:1427298	Sierra de Grazalema, El Bosque, Spain	36° 45′ 08.8″ N 5° 29′ 23.4″ W	^19^
Humerobatidae	*Humerobates rostrolamellatus*		Soil		BMNH:1427572	Sierra de Grazalema, El Bosque, Spain	36° 47′ 26.9″ N 5° 30′ 01.6″ W	^19^
Ceratozetidae	*Ceratozetes peritus*		Soil		BMNH:1427452	Sierra de Grazalema, zona reserva, Spain	36° 46′ 52.4″ N 5° 25′ 39.1″ W	^19^
Phenopelopidae	*Eupelops* sp.		Soil		BMNH:1427516	Sierra de Grazalema, Benamahoma, Las cuevas, Spain	36° 45′ 00.3″ N 5° 25′ 37.9″ W	^19^
Xylobatidae	Xylobatidae sp.		Soil		BMNH:1427453	Sierra de Grazalema, zona reserva, Spain	36° 46′ 52.4″ N 5° 25′ 39.1″ W	^19^
Oribatulidae	*Oribatula tibialis*		Soil		BMNH:1427544	Sierra de Grazalema, Grazalema, molinos harineros, Spain	36° 45′ 59.6″ N 5° 20′ 36.6″ W	^19^
	*Zygoribatula* sp.		Soil		BMNH:1427470	Sierra de Grazalema, zona reserva, Spain	36° 46′ 34.6″ N 5° 26′ 08.5″ W	^19^
Scheloribatidae	*Scheloribates barbatulus*		Soil		BMNH:1427548	Sierra de Grazalema, Grazalema, molinos harineros, Spain	36° 45′ 29.3″ N 5° 21′ 19.0″ W	^19^
	*Scheloribates latipes*		Soil		BMNH:1427523	Sierra de Grazalema, Benamahoma, fuente, Spain	36° 45′ 04.5″ N 5° 26′ 01.2″ W	^19^
Damaeidae	*Metabelba janae*		Soil		BMNH:1427606	Sierra de Grazalema, zona reserva, Spain	36° 46′ 59.3″ N 5° 24′ 41.4″ W	^19^
	*Damaeus flagelifer*		Soil		BMNH:1427501	Sierra de Grazalema, El Bosque, Spain	36° 45′ 21.9″ N 5° 29′ 36.1″ W	^19^
Gustaviidae	*Gustavia* sp.		Soil		BMNH:1427324	Sierra de Grazalema, Benamahoma, Las cuevas, Spain	36° 45′ 00.3″ N 5° 25′ 37.9″ W	^19^
Ceratoppiidae	*Ceratoppia bipilis*		Soil		BMNH:1427607	Sierra de Grazalema, zona reserva, Spain	36° 46′ 59.3″ N 5° 24′ 41.4″ W	^19^
Gymnodamaeidae	*Gymnodamaeus* sp.		Soil		BMNH:1427411	Sierra de Grazalema, Benamahoma, zona recreativa, Spain	36° 45′ 19.8″ N 5° 27′ 12.6″ W	^19^
	*Arthrodamaeus reticulatus*		Soil		BMNH:1427485	Sierra de Grazalema, El Bosque, Spain	36° 44′ 54.0″ N 5° 29′ 25.7″ W	^19^
Pheroliodidae	*Licnoliodes* sp.		Soil		BMNH:1427372	Sierra de Grazalema, Benamahoma, Las cuevas, Spain	36° 45′ 15.5″ N 5° 26′ 36.8″ W	^19^
Oppiidae	*Ramusella* sp.		Soil		BMNH:1427520	Sierra de Grazalema, Benamahoma, Las cuevas, Spain	36° 45′ 00.3″ N 5° 25′ 37.9″ W	^19^
	*Oppia africana*		Soil		BMNH:1427290	Sierra de Grazalema, El Bosque, Spain	36° 44′ 54.0″ N 5° 29′ 25.7″ W	^19^
	*Medioppia* sp.		Soil		BMNH:1427535	Sierra de Grazalema, Grazalema, molinos harineros, Spain	36° 45′ 53.2″ N 5° 20′ 46.2″ W	^19^
Damaeolidae	*Fosseremus quadripertis*		Soil		BMNH:1427412	Sierra de Grazalema, Benamahoma, zona recreativa, Spain	36° 45′ 19.8″ N 5° 27′ 12.6″ W	^19^
Xenillidae	*Xenillus tegeocranus*		Soil		BMNH:1427554	Sierra de Grazalema, Benamahoma, Las cuevas, Spain	36° 45′ 15.5″ N 5° 26′ 36.8″ W	^19^
Hermanniellidae	*Hermanniella granulata*		Soil		BMNH:1427325	Sierra de Grazalema, Benamahoma, Las cuevas, Spain	36° 45′ 00.3″ N 5° 25′ 37.9″ W	^19^
Amerobelbidae	*Mongaillardia* sp.		Soil		BMNH:1427366	Sierra de Grazalema, zona reserva, Spain	36° 46′ 59.3″ N 5° 24′ 41.4″ W	^19^
Cepheidae	*Cepheus latus*		Soil		BMNH:1427590	Sierra de Grazalema, zona reserva, Spain	36° 47′ 42.0″ N 5° 23′ 40.1″ W	^19^
Nothridae	*Nothrus reticulatus*		Soil		BMNH:1427468	Sierra de Grazalema, zona reserva, Spain	36° 46′ 34.6″ N 5° 26′ 08.5″ W	^19^
Camisiidae	*Camisia spinifer*		Soil		BMNH:1427592	Sierra de Grazalema, zona reserva, Spain	36° 47′ 42.0″ N 5° 23′ 40.1″ W	^19^
Crotoniidae	*Heminothrus targionii*		Soil		BMNH:1427334	Sierra de Grazalema, Benamahoma, fuente, Spain	36° 45′ 04.5″ N 5° 26′ 01.2" W	^19^

For each sample, collection date, locality, and eco-type were presented. The last three species belong to the Nothrina and represent the outgroups. Abbreviations for the research institute: *BMNH* Natural History Museum, London, *IBUG* Institute of Biology, University of Graz, *NIPR* National Institute of Polar Research, *NSMT* National Museum of Nature and Science, Tokyo.

Material was sorted and identified under a stereomicroscope. Each genomic DNA was extracted from a single individual using a DNeasy Blood and Tissue Kit (Qiagen), with modifications from Johnson et al.^50^. Specimens were incubated for at least 48 h to lyse the tissue. In the elution step, buffer EB (Qiagen) was used instead of buffer AE to avoid inhibiting the subsequent enzymatic reaction by EDTA.

The total DNAs were quantified by Qubit 4 (Thermo Fisher Scientific) with a dsDNA HS Assay kit. A Collibri ES DNA library Prep kit for Illumina system with UD indexes (Thermo Fisher Scientific) was used to make the shotgun libraries. The amount of input DNA was 30 ng each, and the library target was 300–800 bp. The fragmentation step was set to 37 °C for 10 min. The library amplification was conducted for 12 cycles each. The other procedures were followed by the manufacturer's method. The shotgun libraries were quantified by Qubit 4 and qualified by TapeStation 4200 (Agilent) with a D1000 DNA assay kit.

The indexed libraries were pooled with other libraries, and paired-end sequencing (300 cycles) was conducted by the HiSeq X system (Illumina).

### De novo assembly and annotation

The resulting fastq files were filtered by fastp v. 0.23.2^51^, and sequential processes were conducted by CLC Genomics Workbench v. 12 (Qiagen). The De novo assembly was conducted by default setting, and constructed contigs were selected by BLAST search^52,53^, both implemented in CLC Genomics Workbench.

Annotation for mitochondrial genomes was initially conducted by MITOS2^54,55^, and some protein-coding genes (PCGs) were confirmed manually. Some of the missing tRNAs were searched manually with ARAGORN v1.2.41^56,57^ and RNAfold WebServer^58^. For nuclear rRNA genes annotation, all of the determined sequences were aligned in MEGA7^59^ and determined both side ends of these genes with the aid of RNAfold.

The determined sequences were deposited in DDBJ (DNA Data Bank of Japan, https://www.ddbj.nig.ac.jp) under accession numbers LC817322 –LC817343 (mitogenome) and LC817344-LC817365 (nuclear rRNA).

A range of ca. 6.3–22.5 million raw reads were acquired for these samples in 11.2 million reads on average. After assembling and annotating the raw read, the complete mitochondrial genomes and region, including whole nucleic ribosomal RNA genes (18S, 5.8S, and 28S), were determined for 22 samples. For the determination, the CLC genomics workbench performed de novo assembly, and many contigs were obtained in each sample. From these contigs, we used blast search to select sequences containing the mitochondrial genome and nuclear rRNA genes. We also constructed phylogenetic trees separately to see if there are any significant differences in evolutionary trends between mitochondrial and nuclear genes, which was not the case. The final datasets for mt DNA (13 PCGs) and nucleic rRNA genes were 11,211 bp and 6,920 bp, respectively.

### Phylogenetic analyses

To elucidate the phylogenetic relationship among Brachypylina mites, 25 ingroup and 3 outgroup (Nothrina) mitochondrial genome sequences were obtained from GenBank (Table 2). All these open sequences were without annotation, we conducted gene identification by MITOS2. From total 50 mitochondrial genome sequences, 13 PCGs were aligned in MEGA7 with aid of ClustalW^60^ implemented in MEGA7.

PartitionFinder ver. 2.1.1^61^ was used to determine the best partitioning scheme and substitution model for RAxML-NG^62^ and BEAST 1.10.4^63^ using linked branch lengths and a greedy search algorithm^64^. The optimal partitioning scheme and evolutionary models consisted of thirteen genes data sets for both analyses and were shown in Supplementary Table S2. Bootstrap analyses^65^ of 1000 pseudoreplicates were performed for ML tree.

Also, we reconstructed phylogenetic trees with three nucleic rRNA genes. Each rRNA gene was aligned with MAFFT v7.0.26^66^ using X-INS-i option to consider their secondary structure. Sequential procedures were the same as that of 13 PCGs.

### Phylogenetic dating

Dating was performed using BEAST v1.10.4 software suite. For Molecular clock settings, the dataset was partitioned into 11 partitions (Supplementary Table S2), applying an uncorrelated log-normal clock to each partition. The ML tree generated by RAxML-NG was used for the starting tree. A birth–death speciation prior was applied, and analysis was conducted for 100 million generations with parameters and tree sampling once per 1000 generations.

Two external and two internal calibrations were available for our dataset (Supplementary Table S1). The root calibration prior was set to a normal distribution. Two of the calibration points were set as log-normal distribution. The last internal calibration point was set, ranging 16–1000 by a uniform distribution. The four calibration points were used based on fossil data: the root (310 mya)^5^, the Brachypylina stem group (274 mya)^5^ and crown group (207 mya)^5^ and the family Scutoverticidae stem group (16 mya)^3^ (Supplementary Table S1). Convergence was inferred by Tracer ver. 1.7.2^67^ to ensure that parameter values have an effective sampling size value of > 200. A consensus tree was estimated with TreeAnnotator in BEAST software suite, discarding the first 25% of trees as burn-in.

### Supplementary Information

Supplementary Information.

## Data Availability

Gene sequence data are available at DDBJ/EMBL-Bank/GenBank; accession numbers are given in Table 2 and Supplementary Table S3.
