# Supplementary material for: Mitochondrial metagenomics reveal the independent colonization of the world’s coasts by intertidal oribatid mites (Acari, Oribatida, Ameronothroidea)
Source: Sci Rep. 2024 May 21;14:11634. doi: 10.1038/s41598-024-59423-7 (PMC11109099; doi:10.1038/s41598-024-59423-7)
Supplement: Supplementary file 1 — Supplementary Information. [file 41598_2024_59423_MOESM1_ESM.pdf]

Mitochondrial metagenomics reveal the independent colonization of the world's coasts by intertidal oribatid mites (Acari, Oribatida, Ameronothroidea)

Tobias PFINGSTL, Shimpei F. HIRUTA and Satoshi SHIMANO

Supplemental material

Supplementary Table S1. The list of calibration points for phylogenetic dating analysis in BEAST. The parameters employed in the analysis and also the source are shown.

	Node	Divergence time (mya)	Prior value	distribution	Source
1	Root	310	310±31	Normal	Schaefer et al. (2010)
2	Brachypylina (Crown group)	274	274±29	Log-normal	Schaefer et al. (2010)
3	(Crown group)	207	207±11	Log-normal	Schaefer et al. (2010)
4	Scutoverticidae (Stem group)	16	1000-16	Uniform	Dunlop & Selden (2009)

Supplementary figure S1. Time-calibrated tree inferred from the mitochondrial 13 PCGs dataset. The tree is the same as in Figure 1. Blue bars indicate the 95% confidence intervals for each node's age (mya).

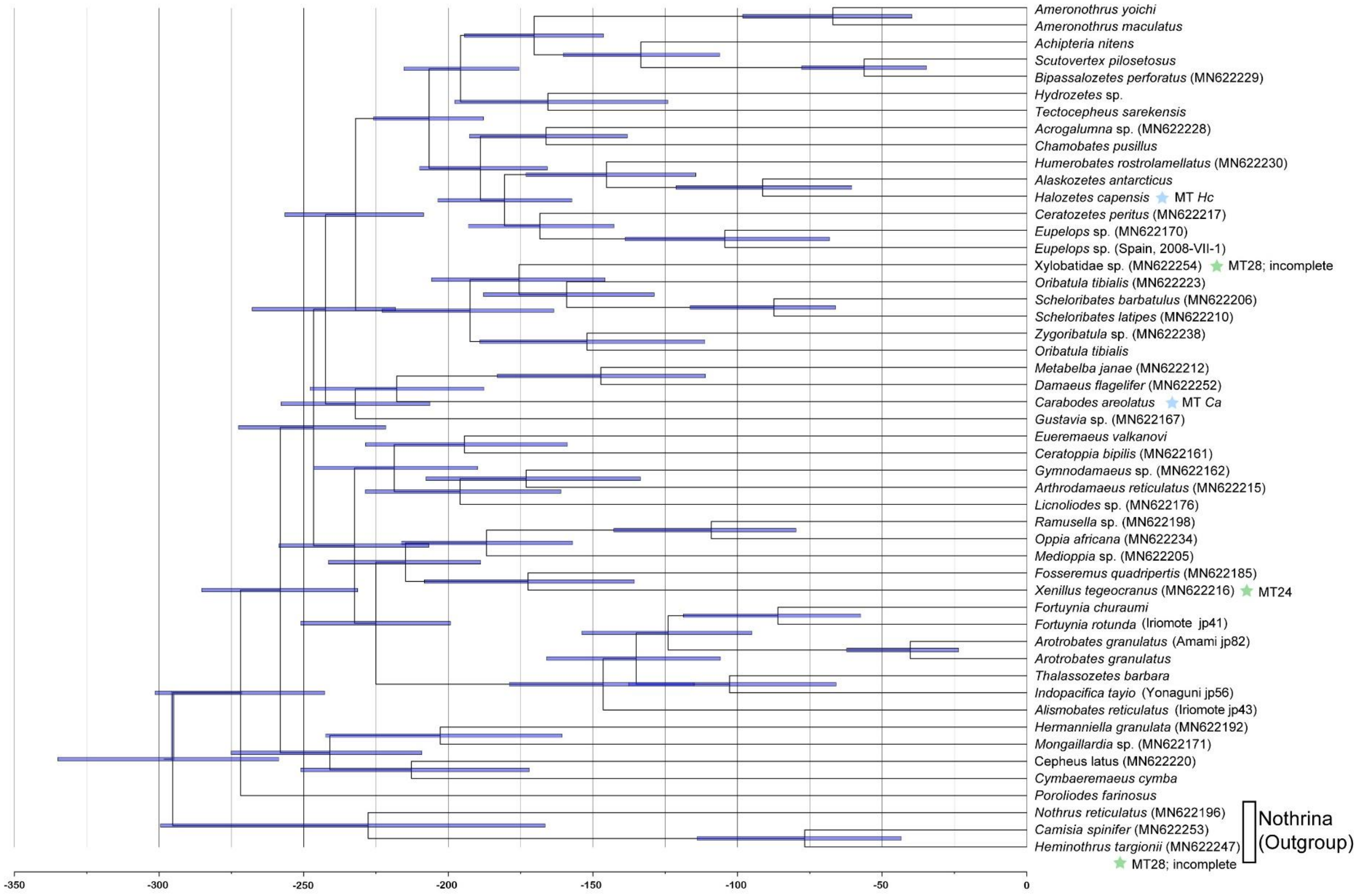

Supplementary Table S2. Best partition schemes and corresponding best models of substitution for 13 PCGs dataset as determined with Partition Finder and subsequently applied in the different analyses.

Partition	model for ML	model for BA	gene	region	
1	GTR+I+G4	GTR+I+G	COX1	0-1566	
2	GTR+I+G4	GTR+I+G	COX2, COX3	1567-2247	3103-3912
3	GTR+I+G4	GTR+I+G	ATP8	2248-2433	
4	GTR+I+G4	GTR+I+G	ATP6, NAD3	2434-3102	3913-4263
5	GTR+I+G4	GTR+I+G	NAD5	4264-5955	
6	GTR+I+G4	GTR+I+G	NAD4	5956-7308	
7	GTR+I+G4	GTR+I+G	NAD4L	7309-7599	
8	TVM+I+G4	HKY+I+G	NAD6	7600-8085	
9	TN93+I+G4	TN93+I+G	CytB	8086-9228	
10	GTR+I+G4	GTR+I+G	NAD1	9229-10176	
11	GTR+I+G4	GTR+I+G	NAD2	10177-11211	

Supplementary Figure S2. Mitochondrial gene arrangement of the Brachypylina mites. Genes highlighted in orange indicate a different location from the Brachypylina general pattern. Grayed-out genes are those that have been undetected or are missing in the sequence. (a) Estimated general pattern of Brachypylina mitogenome. The pattern is identical in the arrangement of PCGs to MitoType 20 in Arribas et al. [19]. Gene arrangement for (b) *Halozetes capensis*, and (c) *Carabodes areolatus*. (d) MitoType 24 (*Xenillus tegeocranus*) and (e) MitoType 28 (Xylobatidae sp. and *Heminothrus targionii*) are annotated results for sequences from Arribas et al. [19].

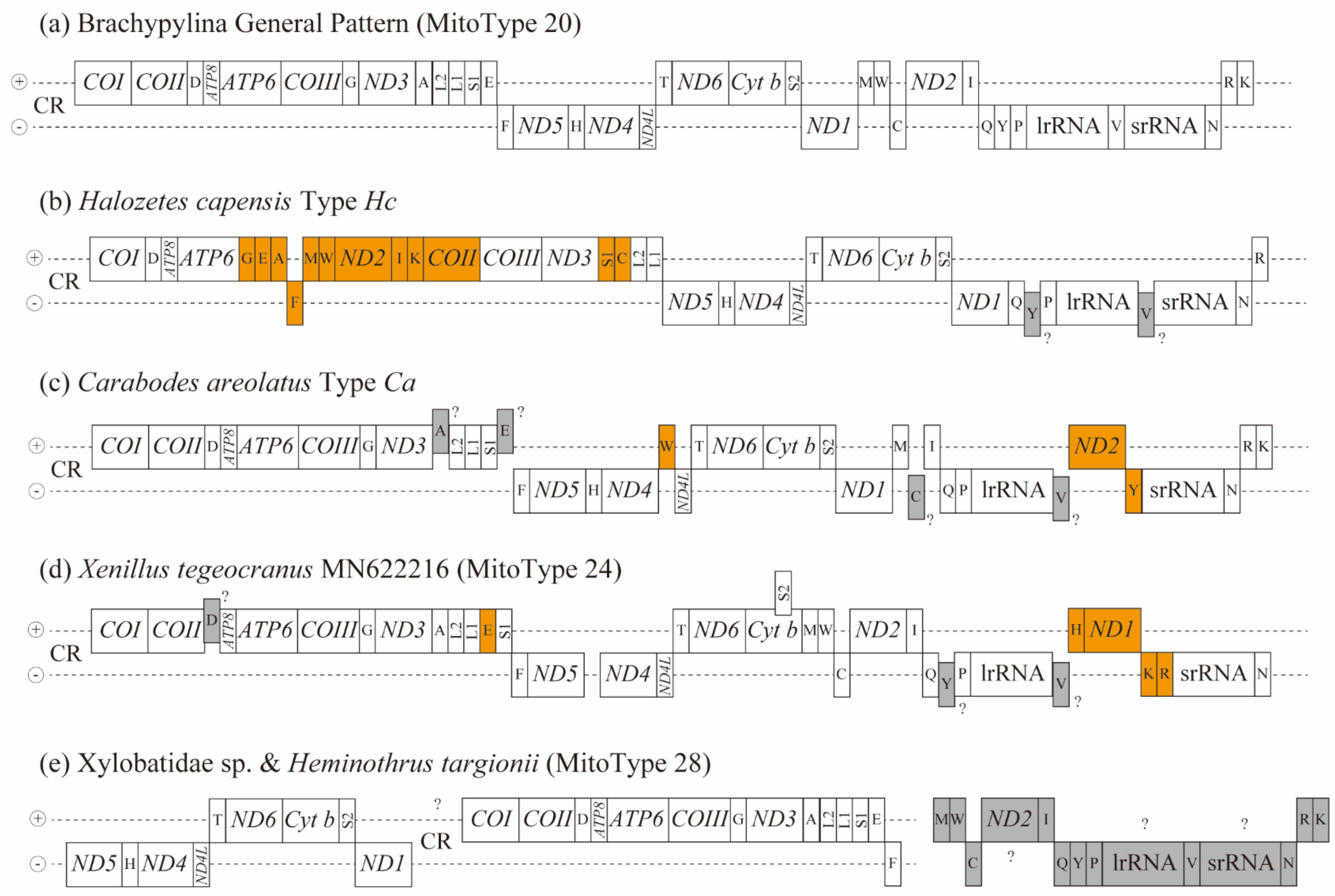

Supplementary Table S3. Annotation summary for the 50 mitochondrial genomes with sequence length and gene arrangement type. The general type of Brachypylina is identical to MitoType 20 [19] in PCGs order. The MitoType 20 with a question mark indicates the sequence is classified as MitoType 20 in [19]; however, it does not include all of the PCGs. The specimens used were shown in table1.

Species	Accession No.	Sequence length (bp)	MitoType	Remarks
Ameronothrus yoichi	LC817322	14 179	General type	trnE, V, and Y not found
Ameronothrus maculatus	LC817323	14 103	General type	trnC, V, and Y not found
Achipteria nitens	LC817324	14 152	General type	trnA not found
Scutovertex pilosetosus	LC817325	13 909	General type	trnH, R, S2, T, V, and Y not found
Hydrozetes sp.	LC817326	14 084	Type *Hc*	trnA and V not found
Tectocepheus sarekensis	LC817327	13 982	General type	trnA, V, and Y not found
Chamobates pusillus	LC817328	14 074	General type	trnA, V, and Y not found
Alaskozetes antarcticus	LC817329	14 245	General type	trnA, F, and V not found
Halozetes capensis	LC817330	15 687	General type	trnV and Y not found
Eupelops sp.	LC817331	14 128	General type	trnV not found
Oribatula tibialis	LC817332	13 993	General type	trnA, R, S1, V, and Y not found
Carabodes areolatus	LC817333	14 178	Type *Ca*	trnA, C, E, and V not found
Eueremaeus valkanovi	LC817334	14 214	General type	trnH and V not found
Fortuynia churaumi	LC817335	14 325	General type	trnA, V, and Y not found
Fortuynia rotunda	LC817336	14 326	General type	trnA, V, and Y not found
Arotrobates granulatus	LC817337	14 333	General type	trnA, V, and Y not found
	LC817338	14 338	General type	trnA, R, V, and Y not found
Thalassozetes barbara	LC817339	14 277	General type	trnA, V, and Y not found
Indopacifica tayio	LC817340	14 348	General type	trnA, I, V, and Y not found
Alismobates reticulatus	LC817341	14 178	General type	trnV and Y not found
Cymbaeremaeus cymba	LC817342	14 947	General type	trnA, M, R, V, and Y not found
Poroliodes farinosus	LC817343	14 447	General type	trnA, H, P, R, V, and Y not found
Bipassalozetes perforatus	MN622229	13 836	20	trnA, H, I, R, S1, V, and Y not found
Acrogalumna sp.	MN622228	14 241	20	trnA, F, H, V, and Y not found

**Supplementary Table S3. Continued**				
Humerobates rostrolamellatus	MN622230	14 060	20	trnA, R, V, and Y not found
Ceratozetes peritus	MN622217	14 087	20	trnA, R, S1, V, and Y not found
Eupelops sp.	MN622170	14 483	20	trnA and V not found
Xylobatidae sp.	MN622254	10 974	28	sequence incomplete
Oribatula tibialis	MN622223	14 542	20	trnA, R, S2, and V not found
Scheloribates barbatulus	MN622206	14 242	20	trnA, C, and V not found
Scheloribates latipes	MN622210	11 456	20?	sequence incomplete
Zygoribatula sp.	MN622238	14 447	20	trnA, L1, and Y not found
Metabelbella janae	MN622212	10 850	20?	sequence incomplete
Damaeus flagelifer	MN622252	10 980	20?	sequence incomplete
Gustavia sp.	MN622167	14 471	20	trnA, C, F, H, and V not found
Ceratoppia bipilis	MN622161	14 763	20	trnA, G, H, M, N, R, and V not found
Gymnodamaeus sp.	MN622162	14 368	20	trnA, E, G, R, and Y not found
Arthrodamaeus reticulatus	MN622215	14 216	20	trnA and V not found
Licnoliodes sp.	MN622176	14 164	20	trnA, C, H, I, R, and V not found
Ramusella sp.	MN622198	13 952	20	trnA and V not found
Oppia africana	MN622234	13 932	20	trnA, S2, V, and Y not found
Medioppia sp.	MN622205	14 112	20	trnA and V not found
Fosseremus quadripertis	MN622185	7 758	20?	sequence incomplete
Xenillus tegeocranus	MN622216	14 806	24	trnA, D, F, S2, V, and Y not found
Hermanniella granulata	MN622192	14 549	20	trnA, F, I, L2, R, S2, and V not found
Mongaillardia sp.	MN622171	14 908	20	trnA, D, M, R, S2, V, and Y not found
Cepheus latus	MN622220	14 683	20	trnA, H, V, and Y not found
Nothrus reticulatus	MN622196	14 549	20	trnA, F, M, N, R, and Y not found
Camisia spinifer	MN622253	11 118	20?	sequence incomplete
Heminothrus targionii	MN622247	11 286	28	sequence incomplete

Supplementary information on mitochondrial gene arrangements:

The mitochondrial sequence lengths of the 22 samples determined by shotgun analysis ranged from 13,909 to 15,687 bp (Supplementary Table S3). The general pattern of mitochondrial genome arrangement in Brachypylina was inferred (Supplementary Figure S2). Two sequences, *Halozetes capensis* and *Carabodes areolatus*, were mutated in the 13 PCGs (protein coding genes) position. The other 20 sequences were general patterns. NAD1 position mutation was found in the published sequence of *Xenillus tegeocranus* (MN622216; MitoType 24 in [19]). On the other hand, the discovery rate for tRNA genes was low, and none of the supposed 22 genes were found in all species. The tRNA genes were relatively variable in their location compared to PCGs among analyzed species.

The general mitochondrial genome gene arrangement in Brachypylina corresponds to MitoType 20 in Arribas et al. [19]. In Brachypylina mites the gene arrangement of their mitochondrial genome is relatively stable. In particular, protein-coding genes and rRNA genes were conservative, but the positions of the other 22 tRNA genes showed minor fluctuations. Among the tRNA genes, Ala (A), Tyr (Y), and Val (V) had low detection rates even when using MITOS2 or ARAGORN, and even if candidate sequences were manually searched using the anticodon sequence at the position estimated from the general type and the secondary structure of sequences were estimated by RNAfold, the detection rate of the appropriate tRNA sequence was usually low. Domes et al. [23] and Schäffer et al. [24] mention that the lack of many tRNAs from the mitochondrial genome of acariform mites is an enigmatic problem and needs further investigation. They also mention, however, that an evolutionary process involving mitochondrial gene reduction is possible. Klimov and OConnor [21] reported very short tRNAs with no D-arm or with short and unstable T-arms. The study said these extraordinary tRNAs are widespread in acariform mites and may also apply to the oribatid Brachypylina mites, which we have sequenced and annotated herein. In the mitochondrial genomes we sequenced, we found different arrangements of major genes in the two species, *Carabodes areolatus* and *Halozetes capensis*. In the mitogenome of *Carabodes areolatus*, NAD2 is transferred between the 16S rRNA and 12S rRNA genes, and several tRNA genes are also moved. In the mitogenome of *Halozetes capensis*, two genes, NAD2 and COII, have moved, and at least 10 tRNA genes have changed their positions. In Arribas et al. [19], the two sequences, of what is considered MitoType 28, are both about 11,000 bp in length and are considered incomplete in that paper. The genes that should be present are indeed missing, and NAD2, 16S rRNA, and 12S rRNA, which are present between COX1 and NAD1 in the Brachypylina mitogenome general pattern, are missing. The registered sequences start from NAD5 and end with the sequential tRNA genes region after NAD3. Considering the sequence of the general type, it is not surprising that the sequence is circular, although there are omissions inside. The sequence was assembled from shotgun sequence data so that it may be an artifact result of the assembler. However, there have been reports of arthropod mitochondrial genomes containing as many as 18 rings [47]. No one can rule out the possibility that the remaining genes are separated into different rings. For the mitogenome of *Xenillus tegeocranus* (MitoType24 in [19]), the protein-coding gene NAD2 and at least four tRNA genes are transferred to different positions.
